# Comprehensive geriatric assessment pilot of a randomized control study in a Swedish acute hospital: a feasibility study

**DOI:** 10.1186/s40814-018-0228-1

**Published:** 2018-01-29

**Authors:** Theresa Westgård, Isabelle Ottenvall Hammar, Eva Holmgren, Anna Ehrenberg, Aase Wisten, Anne W. Ekdahl, Synneve Dahlin-Ivanoff, Katarina Wilhelmson

**Affiliations:** 1 Department of Health and Rehabilitation, Institute of Neuroscience and Physiology, The Sahlgrenska Academy, University of Gothenburg, Arvid Wallgrens backe, House 2, Box 455, 405 30 Gothenburg, Sweden; 2 Department of Occupational Therapy and Physiotherapy, The Sahlgrenska University Hospital, Gothenburg, Sweden; 3 Centre of Aging and Health-AGECAP, University of Gothenburg, Gothenburg, Sweden; 4 School of Education, Health and Social Studies, Dalarna University, Falun, Sweden; 5 Department of Community Medicine and Rehabilitation, Geriatric Medicine, Sunderby Research Unit, Umeå University, Umeå, Sweden; 6 Department of Neurobiology, Care Sciences and Society (NVS), Division of Clinical geriatrics, Karolinska Institute (KI), Solna, Sweden; 7 Department of Clinical Sciences Helsingborg, Lund University, Lund, Sweden; 8 Department of Geriatrics, The Sahlgrenska University Hospital, Gothenburg, Sweden

**Keywords:** Geriatric, Frail older people, Safety, Multidisciplinary team, Occupational therapy, Discharge plan

## Abstract

**Background:**

Comprehensive geriatric assessment (CGA) represent an important component of geriatric acute hospital care for frail older people, secured by a multidisciplinary team who addresses the multiple needs of physical health, functional ability, psychological state, cognition and social status. The primary objective of the pilot study was to determine feasibility for recruitment and retention rates. Secondary objectives were to establish proof of principle that CGA has the potential to increase patient safety.

**Methods:**

The CGA pilot took place at a University hospital in Western Sweden, from March to November 2016, with data analyses in March 2017. Participants were frail people aged 75 and older, who required an acute admission to hospital. Participants were recruited and randomized in the emergency room. The intervention group received CGA, a person-centered multidisciplinary team addressing health, participation, and safety. The control group received usual care. The main objective measured the recruitment procedure and retention rates. Secondary objectives were also collected regarding services received on the ward including discharge plan, care plan meeting and hospital risk assessments including risk for falls, nutrition, decubitus ulcers, and activities of daily living status.

**Result:**

Participants were recruited from the emergency department, over 32 weeks. Thirty participants were approached and 100% (30/30) were included and randomized, and 100% (30/30) met the inclusion criteria. Sixteen participants were included in the intervention and 14 participants were included in the control. At baseline, 100% (16/16) intervention and 100% (14/14) control completed the data collection. A positive propensity towards the secondary objectives for the intervention was also evidenced, as this group received more care assessments. There was an average difference between the intervention and control in occupational therapy assessment − 0.80 [95% CI 1.06, − 0.57], occupational therapy assistive devices − 0.73 [95% CI 1.00, − 0.47], discharge planning −0.21 [95% CI 0.43, 0.00] and care planning meeting 0.36 [95% CI-1.70, −0.02]. Controlling for documented risk assessments, the intervention had for falls − 0.94 [95% CI 1.08, − 0.08], nutrition − 0.87 [95% CI 1.06, − 0.67], decubitus ulcers − 0.94 [95% CI 1.08, − 0.80], and ADL status − 0.80 [95% CI 1.04, − 0.57].

**Conclusion:**

The CGA pilot was feasible and proof that the intervention increased safety justifies carrying forward to a large-scale study.

**Trial registration:**

Clinical Trials ID: NCT02773914. Registered 16 May 2016.

## Background

The examination of frail older patients presenting to the hospital is multifaceted as acute medical problem combined with other ailments make it challenging to find the reason behind the problem requiring admission [1]. This population often requires thorough assessment, continuity, and follow-up [2]. Older people have great confidence in the care and competences that they think hospitals have [3]. Despite this, the health care for older people is often fragmented, focusing on single health complaints and not adequately acknowledging their needs and well-being [4]. A prediction exists that a major focus in the future of in-patient acute medical care is going to be the care of the older people [5]. As the population ages and grows, admittances to the hospital and emergency department (ED) will also continue to increase [6] for older people with chronic and complex illnesses. Adapting health care services by fine tuning the tasks and roles of health care professionals is a supported method that reduces adverse risks during hospitalization while addressing the needs of the frail older people [7].

Frailty includes weakness, fatigue, weight loss, low physical activity, poor balance, slow gait speed, poor motor processing and impaired cognition [8]. Frailty is an age-related central concept, where the reserves and ability to resist stressors presented to the body’s physiological systems are severely limited [9]. Vulnerability in the population with three or more of the frailty factors was found to have a significantly increased risk of hospitalization, disability, and death [8].

Older people who frequently visit the hospital often have social and health care needs [6] and the majority is unable to perform at least one activity of daily living (ADL) which goes unidentified [10]. Hospital programs often do not emphasize the need for geriatric skilled services and staff, despite the increase in geriatric patients requiring hospital admission [11]. Historically, hospital care on acute medical wards is poorly adapted to address the multiple needs of frail older people and is insufficient in providing health care services for problems which could have been readily recognized and treated [12]. This population is frequently exposed to dangers which can exacerbate their loss of function, resulting in unjustified social and health care dependence and even death [3, 13]. A focus on the right approach to health care for older people, stressing the empowerment of older people as rights holders, without discrimination and participation in relation to their health and well-being, is an important role of accountability in health [14].

The World Report on Aging and Health [15] has reported that for older people the maintenance of functional ability has the highest importance. Moreover, World Health Organization (WHO) [15] has identified the needs of older people which must include a transformation of health systems away from disease-based curative models, towards the provision of integrated care. Strategies delivering comprehensive person-centered services to older populations secured in evidence [15] and optimizing opportunities for health, participation, and security in order to improve life as people age [16] can be achieved.

A person-centered method designed for managing the frail population is the Comprehensive Geriatric Assessment (CGA) [7, 17–20], which is a coordination of multidimensional specialties, interdisciplinary diagnostic process used to determine medical, psychological, social, and functional capabilities. The CGA differs from a standard medical evaluation by including diverse domains, where the focus is on restoring healthy function and independence where possible [21, 22]. By aiding in the development of a tailored treatment and follow-up plan, coordination of management of care and evaluation of long-term care needs, enhanced communication, and optimal living conditions [7, 13, 23] are attainable. Finally, the use of a CGA multidisciplinary team on an acute medical ward could produce significant improvements in outcomes of frail older people, including increased survival, improved functioning, and decreased need for elderly care facility placement [2, 18].

Sweden is a country with mainly publicly funded health care, and despite the availability of highly specialized acute care services, Swedish health care is facing challenges related to access, quality, efficacy, and funding [24]. Services are not adapted to address frail older people requiring hospital admission, as older patients are marginalized evidenced by the lack of geriatric competence [25] thus limiting their capabilities and freedom [26]. Frail older people need a combination of approaches which are person-centred in praxis and can allow for their well-being. By identifying and acknowledging the needs unique to the person, the team can better understand and support how to assist and recognize what is required for each person to actively age. In spite of this knowledge, studies are lacking and the literature provides little guidance about the practicalities of implementing and evaluating a CGA program on an acute care setting. An urgent need for assessing the development and implementation of such an intervention in acute hospital care that optimizes the care outcomes of frail older people has yet to be realized [27–30].

This Comprehensive Geriatric Assessment study is designed to examine and prove or disprove if the frail older people who received a CGA intervention could demonstrate improved outcomes in a variety of areas. Specifically, the study will scrutinize independence in activities of daily living, physical function, self-rated health, life satisfaction, quality of care, and health care consumption compared to the control group. Prior to this exploration, a pilot study was carried out to determine the feasibility of the CGA ward’s clinical methods, and research procedures used in the randomized control trial (RCT), before determining if a full-scale study should proceed. This article outlines the original study protocol and explores the feasibility of the pilot implementation for the CGA RCT in a Swedish acute care setting.

### Pilot study objectives

1. The primary objective was to examine the feasibility of the research procedures of the RCT by evaluating the study procedures related to the rates of recruitment, consent, randomization, eligibility, and retention. In addition, determining the feasibility of the data collection form and questions of the CGA research assessment tools by observing completion rates, missing data, and time required to administer the form was analyzed.

2. The secondary objectives were to examine and identify the CGA ward’s clinical methods evidenced through documentation and chart reviews by establish that the intervention participants were assessed in accordance with the CGA domains of functional ability, physical health, psychological state, and social environment. Finally, proof of principle was established for the intervention participants through the surrogate outcomes that they received hospital risk assessments, and care assessments and discharge planning.

### Pilot sample size

Justifying the pilot trial sample size in order to decrease the total sample size of the pilot and the main trial together is described to be the most appropriate means of sample size calculation. Recognition must be paid to the pilot trial, as it is part of a larger clinical study. The nature of this pilot study was to determine the recruitment, retention rates, and proof of principle related to patient safety, and a single point estimate of 30 representative participants was determined [31]. Furthermore, it is the intention of the researchers to include the pilot data in the main study, if sampling and methodologies are the same and feasible [32].

## Methods

As per CONSORT extension for reporting randomized pilot and feasibility trials [33] a pilot study should be conducted to determine the feasibility of the study’s protocol, research procedures, assessment tools, and clinical methods, prior to proceeding with a full-scale RCT. The processes used on the CGA intervention ward were examined for their adherence to the recommendations for evidence-based key objectives for designing pilot and feasibility studies to ensure the methodological design approach taken in the pilot was robust and feasible [33–35]. In order to test that the randomization worked, the intervention and control groups from the pilot were compared at baseline for the participant’s demographics. Furthermore, all baseline data collection questionnaires were reviewed for missing responses, refusals, and data entry anomalies.

## Main study protocol

### Design and setting for main study protocol

The main study is a two-armed design with participants being randomized into a CGA acute geriatric medical intervention group and control acute medical wards in a university hospital in Western Sweden. Eligibility to participate in the CGA study is for persons 75 or older who presents to the ED and require an acute hospital admission, screened as frail using the FRESH-screen [36] and not admitted via fast track (for stroke, coronary infarct, or a hip fracture).

### Intervention group

The participants randomized to the intervention group receive CGA which involves specialized treatment on a geriatric acute medical ward. Assessments are both comprehensive and person-centered, and are provided by an multidisciplinary team to address the frail older people’s multiple needs as they related to physical health, functional ability, psychological state, cognition, and social environmental circumstances [2, 7]. The focus of person-centeredness is to consider the social world of the person with regards to their everyday life, relationships with others and belief system as it narrates personal values, goals, and motivations [37]. A person-centered approach is tailored by the operational CGA team which consisted of a medical doctor, nurse and nurse assistant (NA), occupational therapist (OT), and physical therapist (PT). When appropriate, the team is extended to include a social worker and a nutritionist.

The key components of the CGA team is to essentially optimize the care and well-being of the frail older patients after identifying their multidimensional and rehabilitation needs, while putting forth a discharge plan in partnership with the frail older person which included recommendations for long-term follow-up. The assessments used on the intervention ward to safeguard a comprehensive understanding of all health domains, enabling problems of the frail older person’s problems to be identified and coordinated by the multidisciplinary team are described in Table 1.

**Table 1:** Clinical assessment tools (CGA team on the intervention ward)

Domain	Clinically assessed	Profession
Functional ability: person’s ability to execute tasks that are essential for living	ADL status	OT
	Balance and mobility	PT
	Risk of fall	PT
Physical health: comprehensive medical and polypharmacy history, fall risk, pain, nutrition, decubitus ulcer, elimination, and oral hygiene	Illness rating	Physician
	Medication review	Physician
	Risk assessment	Nurse/NA
	Risk for fall	Nurse/NA
	Nutrition	Nurse/nutritionist
	Decubitus	Nurse/NA
Psychological state: screen for depression, cognition, and delirium	Risk assessment	Nurse/NA
	Depression (p.r.n.)	Physician
	Cognition (p.r.n.)	OT
	Delirium (p.r.n.)	Physician/OT
Social environment: needs and support	Social needs and support network	Nurse/social worker

*OT* occupational therapy, *PT* physical therapy, *CGA* comprehensive geriatric assessment, *NA* nurse assistant, *p.r.n.* pro re nata

### Control group

The participants randomized to the control group receive treatment on the acute medical wards. Several of the nursing and PT staff working on the control ward has geriatric competence and training from previous professional experience. Treatments and services such as those provided by OT, PT, social work, and the nutritionist are not automatically included on the acute medical wards; rather referrals are required from physicians or nursing, if participants requires a consultation, assessment, and or treatment from these disciplines.

### Recruitment, consent, and randomization

Eligible candidates for the study are identified in the ED by the care coordinator (a nurse assistant). This individual is responsible for recruitment and the randomized inclusion, which is possible only if beds are available on both the inclusion and control wards. Prior to inclusion into the study, potential participants are invited to join. They are informed about the study, how it is conducted, what is expected of them, and that participation is voluntary. Information is provided both in writing and verbally. An opportunity to ask questions is offered. If they agree to participate, a consent form is signed by the participant. Following consent, the randomization is done by a computer-generated numbers and assigned by the case coordinator, where the allocation concealment with a sequentially numbered opaque sealed envelope (SNOSE) is employed. Due to the complexity of the ED including the turnover of staff, shifts, and high pace, it is not viable that an additional person be assigned in safeguarding the randomization.

### Study sample size and calculated power for the main study

A power calculation has been created based on the primary outcome variable, dependence in activities of daily living (range 0–9), with an assumed difference between the intervention and control groups of one dependence (i.e., dependent in one more activity of daily living, a clinically relevant difference of importance to the individual as well as the caregiver), and a standard deviation of 2 in both groups. To detect a difference between the intervention and control groups with a two-sided test and with a significance level of α = 0.05 and 80% power, at least 64 participants in each group are needed. To account for loss to follow-up, a total of 156 persons (78 in the control group and 78 in the intervention group) will be included in the study. The power calculation and the assumed loss to follow-up, of 22%, are based on previous research on frail older people [38]. Furthermore, this study intends to pool the pilot and main study data, if the methods, procedures, and data collection remain the same following the pilot study.

### Data collection

Once transferred to the ward and prior to the baseline interview, the researcher does a chart review and then completes the data collection with the participants on their respective wards (intervention and control) using the research assessment tools, see Table 2.

**Table 2:** Assessment tools (research team)

Domain	Research assessment/tools	Outcome
Functional ability: person’s ability to execute tasks that are essential for living	ADL staircase [45]	Primary
	Berg’s balance scale [42]	Secondary
	Timed up and go (TUG) [46]	Secondary
	Gait speed [41]	Secondary
	Fall efficiency scale-international (FES-I) [47]	Secondary
	Dynamometer [39]	Secondary
Physical health: comprehensive medical history fall risk, pain, nutrition, elimination, and vision	Cumulative Illness Rating Scale-Geriatric (CIRCS-G) [48]	Secondary
	Gothenburg Quality of Life Instrument (GQL) [49]	Secondary
	Self-rated health	Secondary
	KM visual acuity chart [40]	
	Health care consumption: register data	
	Mortality	Secondary
Psychological state: screen for confusion, depression and cognition	Mini Mental State Examination (MMSE) [43]	Secondary
	Montgomery–Åsberg depression rating scale (MADRS) [50]	Secondary
	Capability measure for older people (ICECAP-O) [51]	Secondary
	Fugl-Meyer-Lisat 11 [52]	Secondary
Social environment: needs, support, and network	Impact on Participation and Autonomy (IPA-O) [53]	Secondary
	Satisfaction with quality of care	Secondary
	Home health care	Secondary
	Informal care	Secondary

The participants are systematically followed up per study protocol in the person’s home (or place of discharge), 1 month, 6 months, and 12 months after discharge from hospital. When possible, researchers are blinded during the 1-month data collection for both the intervention and control groups. Furthermore, the participants in the study are also blinded during the RCT and do not know if they are on a control or intervention ward. Finally, the staff is not informed that the patients they are treating are included in the study. The interviews and instruments employed are the same during all phases. Participants are contacted via telephone by the researchers and home visits are scheduled. In cases where participants are unable to complete the study, proxy questions are used, if approved by the participants. All data collected during the RCT are entered into a password-protected database for baseline, 1, 6, and 12 months. The original paper documents are filed in binders and stored in a locked study office.

### Key research outcomes, domains, and assessments for the main study

The primary outcome measure is dependence in activities of daily living. Secondary outcome measures are capability, self-determination, physical function, self-rated health, life satisfaction, morbidity, symptoms, depression, cognition, satisfaction with quality of care, health care consumption, formal care, and mortality. More information on the assessments used for the primary and secondary outcome measures can be found in Table 2.

Furthermore, frailty phenotypic criteria indicated is comprised of eight factors, which are assessed separately using instruments and questions to address weakness: hand dynamometer [39], vision: KM visual acuity chart at 1 m [40], gait speed: walking four meters at a speed of 6.7 s or slower [41], balance: Berg’s balance test [42], cognition: MMS-E [43], weight loss: question if they have lost weight in the past 3 months, fatigue: question if they have suffered any general fatigue/tiredness over the last 3 months, and physical activity: was defined as one to two walks per week or less [44].

### Statistical analysis for the main study

Both descriptive and analytical statistics will be used, in order to compare groups and for analyses of changes over time using IBM SPSS Statistics for Windows, Version 24.0, 2016, Armonk, NY: IBM Corp. Non-parametric statistics will be used when ordinal data are analyzed. Otherwise, parametric statistics will be used. Besides descriptive statistics, the chi^2^ and Fisher’s 2-tailed exact test to test differences in the proportions between the groups will be used. A value of *p* ≤ 0.05 (2-sided) will be considered significant. Analyses will be made on the basis of the intention-to-treat principle, meaning that participants will be analyzed on the basis of the group to which they were initially randomized. Given the old age of the frail participants, a relatively high drop-out and or death rate is inevitable. The pattern of ‘missingness’ is described as non-random, since the likelihood that a missing response is directly related to data that were collected or requested [54]. Simply analyzing complete cases will not be relevant and might lead to bias about treatment effects. Therefore, a model addressing data imputation for missing data will be employed which replaces missing values with a value based on the median change of deterioration (MCD) between baseline and follow-up [55]. The reasons for this imputation method is (1) the study sample (frail older people) is expected to deteriorate over time as a natural course of the aging process and (2) reasons for not fulfilling the follow-ups are often deteriorated health. Worst-case changes will be used for those who have died before follow-up.

### Statistical analysis for the pilot study

Both descriptive and analytical statistics were used to compare between the intervention and control group. Independent samples *t* test was performed using IBM SPSS Statistics for Windows, Version 24.0, 2016, Armonk, NY: IBM Corp and are reported as mean difference with a 95% confidence intervals (CI).

## Results

### Pilot baseline characteristics

**Fig. 1:**
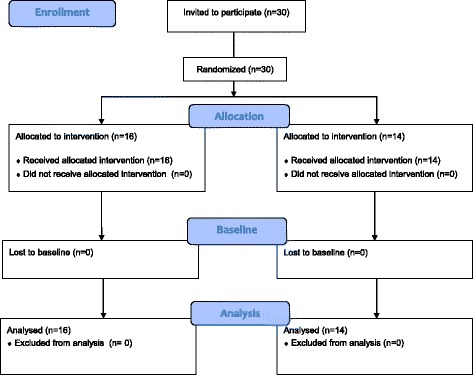
CONSORT 2010 flow diagram for the CGA pilot study

Of the 30 participants asked to participate, all consented. See Fig. 1 for the CONSORT 2010 flow chart showing the inclusion and randomization.

The ages ranged from 77 to 96 and 60% were female, 57% lived alone and 10% had higher education. Forty-three percent had decreased cognitive status and 100% were screened as frail. Additional information related to the participants can be found in Table 3.

**Table 3:** Demographic characteristics of pilot participants at baseline

	Intervention *n* = 16	Control *n* = 14
Age range (mean)	77–96 (86)	77–93 (86)
Female, *n* (%)	9 (56)	9 (64)
Living alone, *n* (%)	9 (56)	8 (47)
Education*, *n* (%)	2 (13)	1 (7)
Impaired cognition**, *n* (%)	7 (44)	6 (43)
Screened for frailty***, *n* (%)	16 (100)	14 (100)
Two frailty factors, *n* (%)	2 (25)	2 (29)
Three frailty factors, *n* (%)	8 (50)	5 (36)
Four frailty factors, *n* (%)	6 (38)	5 (36)
Decreased endurance, *n* (%)	16 (100)	12 (86)
Tired (last 3 months), *n* (%)	11 (69)	13 (93)
Fall tendency/fear of falling, *n* (%)	11 (69)	7 (50)
Help with grocery shopping, *n* (%)	13 (81)	10 (71)

*Tertiary education (university or college)

**Mini Mental State Exam [43], (<25)

***FRESH-screen [36], (decreased endurance, tired (last 3 months), fall tendency, help with grocery shopping)

### Clinical methods

Common elements unique to the CGA practices and the team approach encompassed tailored treatment, focus on discharge planning and follow-up, and practiced good communication (both written and verbally) with fellow team members, during daily rounds and with the participants on the ward. On a weekly basis, rounds on the intervention wards comprised two additional team members addressing the comprehensive needs of those requiring nutrition and social work services.

### Proof of principle

Chart reviews were performed for all 30 participants comparing the 16 on the CGA intervention ward, with the 14 on the medical control wards to further examine the “proof of principle”. It was found that a structured risk assessment to a higher extent was documented among the intervention group which was statistically significant compared to the control group in addressing and documenting the risk for falls, nutrition, decubitus ulcers, and ADL status. See Table 4. Furthermore, it was noted following the chart reviews that the staff working with the control group occasionally addressed safety but were not consistent or systemic in how they documented this information in the charts.

**Table 4:** Structured safety risk assessment documented on wards

	Intervention *n =* 16	Control *n =* 14	Mean difference (CI)*
Risk for falls, *n* (%)	15 (94)	0 (0)	− 0.94 (− 1.08,-0.80)
Nutrition, *n* (%)	15 (94)	1 (7)	− 0.87 (− 1.06,-0.67)
Decubitus ulcers, *n* (%)	15 (94)	0 (0)	− 0.94 (− 1.08,-0.80)
Mobility and transfer status, *n* (%)	13 (81)	10 (71)	− 0.10 (− 0.42, 0.23)
ADL status, *n* (%)	14 (88)	1 (7)	− 0.80 (− 1.04,-0.57)

*95% confidence intervals (CI)

Despite demographic characteristic similarities between the intervention group and the control group, see Table 3, additional chart reviews revealed variability amongst the participants regarding the health care services received on the wards. In part, the pilot study intended to secure that the intervention ward was practicing and documenting as a CGA proof of principle. Following chart reviews of all 30 pilot participants, occupational therapy services were more often received and were statistically significant for the intervention group compared with the control. See Table 5.

**Table 5:** Team members and services received on wards

	Intervention *n =* 16	Control *n =* 14	Mean difference CI**
Medical doctor, *n* (%)	16 (100)	14 (100)	–
Nursing, *n* (%)	16 (100)	14 (100)	–
OT assessment, *n* (%)	14 (88)	1 (7)	−0.80 (−1.06,-0.57)
OT assistive devices*, *n* (%)	14 (88)	2 (14)	−0.73 (−1.00,-0.47)
PT assessment, *n* (%)	13 (81)	10 (71)	−0.10 (−0.42, 0.23)
PT assistive devices*, *n* (%)	13 (81)	10 (71)	−0.10 (−0.42, 0.23)
Nutritionist, *n* (%)	3 (19)	1 (7)	−0.12 (−0.38, 0.14)
Social Worker, *n* (%)	1 (6)	0 (0)	−0.63 (−0.20, 0.08)
Discharge plan, *n* (%)	16 (100)	11 (79)	−0.21 (−0.43, 0.00)
Care planning meeting***, *n* (%)	8 (50)	2 (14)	−0.36 (−0.70,-0.02)

*Assistive devices entails: needs assessment, training with, arranging for use of on the ward and or at discharge

**95% confidence intervals (CI)

***Care planning meeting: ward staff, municipality home health services and patient planning of services required after discharge

### Recruitment, consent, and randomization during pilot study

Inclusion for the study was estimated at three per week, and the pilot was projected to take 10–12 weeks. However, the pilot study took 32 weeks to complete the inclusion. During portions of the pilot, admission to hospital wards had an estimated wait time of up to 48 h, and in some cases, patients were treated and discharged from the ED without ever receiving a bed or reaching a ward. The inclusion process was frequently retarded, due to the lack of available beds on the acute medical wards. Additional control wards were opened during the pilot study to increase inclusion rates.

One hundred percent of participants were screened for frailty; however, the majority (29/30) were screened after inclusion and randomization and 100% (30/30) met the FRESH-screen [36] criteria for frailty, see Table 3. In the early stages of the pilot, it was identified that not all participants were screened with FRESH-screen [36] in the ED prior to inclusion in the study. Furthermore, it was confirmed by ED staff that the FRESH-screen [36] was used more routinely by staff responsible for discharging patients from ED, but not by those responsible for admitting patients to the medical wards. Understanding and accepting that the tools use was not yet a routine among staff, this problem was rectified by adding the FRESH-screen [36] to the baseline questionnaire used by researchers to confirm that participants met the criteria for frailty and met the inclusion criteria for the study.

Consent was 100%, as all participants asked to participate before randomization and transferring to a medical ward agreed. Randomization was used to ensure that each arm of the study would be equal by employing a simple randomization to ensure a balance of the treatment groups with respect to the various combinations of the prognostic variables.

### Retention rates

When the pilot study closed after 32 weeks, four (13%) participants had quit the study, two from the intervention, and two from the control group.

### Research data collection

The baseline interviews took between 25 and 160 min, with a median time of 102 min. While it was understood, that the clinical assessment tools employed by the researchers were numerous, the comprehensive nature of this study at baseline was steadfast. Despite the wide range in time required to complete the baseline, researchers when necessary completed the data collection as piece work, due to the participants being too tired, too ill, and due to higher priority hospital procedures taking precedence. Therefore, data collection often required two, three, and on occasion four visits to the participants to complete the assessments.

Following the pilot, data was examined by performing chart reviews and comparing the data forms with what was entered into the data base. Clarifications explaining “incomplete data and refusals” were documented as comments on the baseline forms by the researchers for participants who were too ill, too tired, too occupied with others (i.e., medical personnel, lab tests, X-rays), or just simply refused to participate. On one occasion during the pilot, a participant was discharged prior to the completion of their baseline. The research team discussed this dilemma and rather than losing the individual in the study, the baseline was completed in the home, as soon as possible after discharge.

Throughout the pilot, the research team discussed their observations about how the participants acted during the assessment, often describing them as fatiguing quickly, refusing certain tests, and or not wanting to continue with the study after the baseline or 1-month follow-up. As a remedy, researchers reorganized the order of assessments to stagger the physical tests, allowing for recovery following physically exerting assessments and at the 1- and 6-month follow-ups the number of assessments and questions were reduced. Furthermore, during the pilot not all participant could speak Swedish and it was discovered that two test were culturally biased as the participant was required to read the Latin alphabet. This was addressed and rectified in subsequent follow-ups using universal symbols (for the KM vision test), and the translator writing out the text in the native language (for the MMSE).

The study was originally planned with the intention to use proxy questions if participants decided that they could not complete the study; however, proxy questions were never utilized during the pilot. Rather it was decided interviews for those with decreased cognitive status could be completed with the support of a next of kin, rather than having incomplete data or losing the participant. Furthermore, during the pilot the research team identified the need for flexibility in the data collection procedures, as a means to keep people in the study. It was agreed upon that a shortened version of the questionnaire would be permitted to use over the telephone, for those not permitting a home visit and the primary outcome measure for the main study the ADL staircase, was prioritized. In addition, several secondary outcome measures addressing physical health, fatigue scale, psychological state, self-rated health question, and social environment were prioritized, as well if there had been changes to home help/home health services. This method was applied on two occasions during the pilot study, once at the first month follow-up and once at the sixth month follow-up. The telephone interviews lasted 10 and 8 min, respectively. See Table 6 for a summary of pilot adjustments.

**Table 6:** CGA adjustments following the pilot

	Original protocol	Adjustments after pilot
FRESH-screen	Screen in ED	Screen in ED or during baseline
Control ward	One	Three
Inclusion	Three per week	Two per week
Follow up assessments	Home visit	Telephone interview p.r.n.
Baseline	Complete on ward	Complete at place of discharge p.r.n.
Data collection	Complete in entirety	Allow for incomplete data/refusal
		Complete as piece work if required
Assessment and questionnaire	Organized by category	Reorganized by importance of domain
		Stagger physically exerting tests
Length of questionnaire	Full assessment: baseline, 1, 6, and 12 months	Condense 1- and 6-month follow-ups
Questions and language	Swedish and Latin alphabet	Use translators and symbols if required

*ED* emergency department, *p.r.n.* pro re nata

## Discussion

The preliminary evidence in the pilot as hypothesized emphasized a positive predisposition towards those receiving intervention provided on the CGA ward. This was confirmed by chart reviews and documentation which displayed that they were receiving health care services with increased focus on safety and were assessed and treated by a multidisciplinary team which was statistically significant when including an OT. Furthermore they had a greater tendency to receive a care plan and leave the hospital with a discharge plan compared to the control group. With regards to a nonsignificant difference in PT services on the wards, it is thought to be attributed to geriatric competent and trained staff that delivered services to the frail older people regardless of their ward or intervention intent. In this framework, noteworthy attention is warranted in the organization and working procedures on an acute medical ward for geriatrics. Examining the process of screening for frailty, safety, clinical assessments, and common elements related to communication and planning crucial to the CGA team intervention is essential. The process of working comprehensively to assess all health domains, regardless of reason for admission onto the acute medical ward [7], and increasing interest in optimizing strategies for delivering comprehensive person-centered health care services to the older population is viable.

The pilot study provided an indication of the rates of recruitment, refusal, eligibility, retention, and sample size which should be expected in a full-scale RCT. Despite the pilot’s success, a noteworthy limitation was the slow inclusion rate, predominantly complicated by the reduction in the number of beds at the hospital [56] cut backs [24, 57] and a systemic shortage of nursing staff [58] which decreased the possibility to randomize people eligible for inclusion. While the pilot study highlights the weakness, the organizational and operational malfunction of the hospital could not be controlled or adjusted by the researchers. Politicians and hospital administrators through negotiations have addressed and attempted to resolve this predicament stymieing the hospitals organization. Efforts to amend the overcrowding in the ED were addressed by making more beds available on the hospital wards. The research team was proactive and added additional control wards increasing the likelihood for randomization and expedited the inclusion rate. Although the issue remains unresolved, it was evaluated following the pilot and a new estimate of two inclusions per week has been deemed feasible for this RCT going forward.

Furthermore, several limitations were discovered in the pilot related to screening, recruitment, and randomization procedures. Specifically, the majority of FRESH-screen (securing that participants were frail) was done by researchers after the inclusion and randomization. The pilot plan was designed that people (75 or older) should be screened for frailty when presenting to the ED [10, 15, 36, 59]; however, this was proven not to be the case in current practice procedures. Fortunately, all of the RCT participants later screened met the criteria as frail, but this was a concern during the pilot. To safeguard the FRESH-screen frailty inclusion criteria, screening was added during the pilot, to the researcher’s protocol prior to the baseline data collection. Furthermore, a limitation might be that the pilot study results were not used to calculate the sample size for the main study. The pilot study aim was not to assess the output from ADL, but rather to insure randomization and retention and its surrogates regarding risk assessments and safety*.*

Another limitation identified in the pilot was related to the recruitment situation, as the responsibility to minimize bias was identified. Specifically, the person with access to the SNOSE should be distinct from those recruiting the participants into the study [60]. However, an acceptation to this rule as in the case of this pilot’s procedure for recruitment and randomization, which is centered on emergency medicine and the emergency department, can be deemed feasible if employing the SNOSE [60]. Initial estimates of the demographics obtained in this pilot study were confirmed during the randomization process, and the retention rates are within the forecasted range set for the full RCT.

Regarding the assessment, it has been observed that completing the baseline was time-consuming due to comprehensive nature of this study. However, researchers made adaptations by reorganizing the sequence of questions prioritizing by domain and allowing for rest between physically exerting tests and completing the data collection as piece work if necessary. However, the baseline questions remained steadfast. Consequently, the 1- and 6-month follow-up forms were amended, reducing the number of clinical assessments/questions, to better accommodate the frail older participants. The 12-month follow-up however was unchanged and mirrors the baseline, with measures in place for missing data using MCD if necessary.

Finally, when examining and identifying the CGA ward’s clinical methods, a proof of principle has been highlighted in the pilot by the practices on the intervention ward with good confidence. With regards to the research procedures and in determining the feasibility, it was found that the pilot study data collection had incomplete data and refusals. Many of these issues were clarified due to the ill and or frail state of the participants as assessments were omitted, and or not completed, in their entirety during the data collection. Other issues related to the procedures are clarified in Table 6.

## Conclusions

The pilot has proven to be feasible in its methods, procedures, and data collection processes and thus is deemed secure in carrying forward to a large-scale study. The proof of principle supporting a CGA intervention was statistically significantly associated with risk assessments and occupational therapy services, and a positive tendency for receiving a care planning meeting and discharge plan compared to the control group. The pilot data is reasoned to be valid and will be utilized and pooled with the large-scale study data. This CGA randomized control trial is the precursor which may identify a breach in Swedish acute medical wards when treating the frail older population. Identifying and measuring what matters and sharing the information in a comprehensive manner will support health care workers’ competence and behavior towards people and helps to build trust. Furthermore, the values and goals unique to the frail older person are the basis for the approach securing active aging. Finally, by using the CGA model opportunities for health, participation and security are optimized, enhancing life as people age and focusing on what people themselves value. Refining and transforming the health system’s methods, standards, and policies away from disease-based curative models towards the establishment of person-centered integrated care, as displayed in the pilot, could have significant implications for the future of frail older people’s health care.

## Abbreviations

ADL: Activities of daily living
CGA: Comprehensive Geriatric Assessment
CI: Confidence intervals
CIRCS-G: Cumulative Illness Rating Scale for Geriatric
CONSORT: Consolidated Standards of Reporting Trials
ED: Emergency department
FES-I: Fall efficiency scale-international
FRESH: Frail elderly support research group
GQL-Instrument: Gothenburg Quality of Life Instrument
ICECAP-O: Capability measure for older people
IPA-O: Impact on participation and autonomy-Older persons
KM: Konstantin Moutakis
MADRS: Montogomery–Åsberg depression rating scale
MCD: Median change of deterioration
MMS-E: Mini Mental Status Examination
NA: Nurse assistant
OT: Occupational therapist
p.r.n.: Pro re nata
PT: Physical therapist
RCT: Randomized controlled trial
SNOSE: Sequentially numbered opaque sealed envelope
TUG: Timed up and go
WHO: World Health Organization
